# Cross-cultural adaptation and psychometric validation of point-of-care outcome assessment tools in Chinese palliative care clinical practice

**DOI:** 10.1186/s12904-024-01395-6

**Published:** 2024-04-03

**Authors:** Yunyun Dai, Claire E Johnson, Jinfeng Ding, Yongyi Chen, Alanna Connolly, Lianjun Wang, Barbara A Daveson

**Affiliations:** 1 Faculty of Science, Medicine and Health, University of Wollongong, 239 Squires Way, Wollongong, 2522 New South Wales Australia; 2 School of Nursing, Guilin Medical University, Guilin, Guangxi China; 3 Palliative Aged Care Outcomes Program, Faculty of Science, Medicine and Health, University of Wollongong, Wollongong, New South Wales Australia; 4 Xiangya School of Nursing, Central South University, Changsha, Hunan China; 5 Hunan Cancer Hospital, The Affiliated Cancer Hospital of Xiangya School of Medicine, Central South University, Changsha, Hunan China; 6 Palliative Care Outcomes Collaboration, Faculty of Science, Medicine and Health, University of Wollongong, Sydney, New South Wales Australia

**Keywords:** Palliative care, Point of care outcomes assessment, Symptom assessment scale, Palliative care problem severity score, Palliative care phase, Validity, Reliability

## Abstract

**Background:**

A standardized national approach to routinely assessing palliative care patients helps improve patient outcomes. However, a quality improvement program-based on person centered outcomes within palliative care is lacking in Mainland China. The well-established Australian Palliative Care Outcome Collaboration (PCOC) national model improves palliative care quality. This study aimed to culturally adapt and validate three measures that form part of the PCOC program for palliative care clinical practice in China: The PCOC Symptom Assessment Scale (PCOC SAS), Palliative Care Problem Severity Scale (PCPSS), Palliative Care Phase.

**Methods:**

A study was conducted on cross-cultural adaptation and validation of PCOC SAS, PCPSS and Palliative Care Phase, involving translation methods, cognitive interviewing, and psychometric testing through paired assessments.

**Results:**

Cross-cultural adaptation highlighted the need to strengthen the link between the patient’s care plan and the outcome measures to improve outcomes, and the concept of distress in PCOC SAS. Analysis of 368 paired assessments (*n* = 135 inpatients, 22 clinicians) demonstrated that the PCOC SAS and PCPSS had good and acceptable coherence (Cronbach’s a = 0.85, 0.75 respectively). Palliative Care Phase detected patients’ urgent needs. PCOC SAS and PCPSS showed fair discriminant and concurrent validity. Inter-rater reliability was fair for Palliative Care Phase (k = 0.31) and PCPSS (k = 0.23–0.30), except for PCPSS-pain, which was moderate (k = 0.53).

**Conclusions:**

The Chinese version of PCOC SAS, PCPSS, and Palliative Care Phase can be used to assess outcomes as part of routine clinical practice in Mainland China. Comprehensive clinical education regarding the assessment tools is necessary to help improve the inter-rater reliability.

**Supplementary Information:**

The online version contains supplementary material available at 10.1186/s12904-024-01395-6.

## Introduction

Point-of-care, outcome assessments help to identify and address the holistic needs of palliative care patients and their families/carers [1, 2]. These clinical assessments enable healthcare professionals to tailor interventions and support individual patients, resulting in improved patient outcomes and enhanced quality of care [1, 3]. Selecting tools that are sensitive and comprehensive for routine clinical use is crucial for accurately identifying holistic palliative care needs [4].

Collecting nationally agreed-upon point-of-care outcome assessments and systematic feedback on health services enhances the understanding of quality, safety, and outcomes of care [5], and it can help build national capacity in palliative care. This is important in the context of China’s development of palliative care, given its aging population and increasing incidence of cancer and chronic diseases [6]. However, there remains a lack of standardized assessment tools tailored to meet the unique needs of palliative care patients and can be effectively and feasibly used for bedside assessment in routine clinical practice. Furthermore, the absence of a national standardized quality improvement program-based on bedside assessments for palliative care in Mainland China also warrants attention. Introducing of a mature, effective, and feasible quality improvement program could ensure the quality of palliative care, particularly during the early stage of its development in Mainland China.

An exemplary national initiative that has demonstrated statistically and clinically significant improvements in patient outcomes, is the Palliative Care Outcomes Collaboration (PCOC) [1, 7], which including two core components. One central part to PCOC is embedding point-of-care assessments and a response framework into routine clinical practice (Supplementary Fig. 1) [8]. The palliative care needs of patients are assessed using five standardized tools, including the PCOC Symptom Assessment Scale (PCOC-SAS) [9] for symptom distress, Palliative Care Problem Severity Score (PCPSS) [10] for symptom severity, Palliative Care Phase [11] for clinical acuity and urgency, the Australia-modified Karnofsky Performance Status (AKPS) [12] for performance status, and the Resource Utilization Groups - Activities of Daily Living (RUG-ADL) [13] for functional dependency. In Australia, these assessments are regarded as the “vital signs” for palliative, providing accurate and dynamic insights into patients’ care needs, enabling clinicians to plan and deliver holistic care accordingly [1, 14]. The additional component of the PCOC program is the production of a quality report based on patients’ clinical outcomes, compared with the national quality benchmarks, aeras need to be improved will be identified, with improvements supported by improvement facilitators across the country and benchmarking workshops. Please see the PCOC model cycle in Supplementary Fig. 2.

Given the feasibility, effectiveness, and success of the PCOC model internationally, we plan to adopt the PCOC model in a Chinese cancer hospital, and then scale up nationally. Before formally integrating the PCOC tools and response framework, it’s important to validate the PCOC assessment tools in the Chinese context. Therefore, this study aimed to cross-culturally adapt the PCOC SAS, PCPSS, and Palliative Care Phase and assess their validity in routine practice in mainland China.

## Methods

The cross-cultural adaptation component of the study included forward and backward translation of the tools, and cognitive interviewing to ensure the quality of cross-cultural adaptation. The validation component of the study involved psychometric testing.

### Cross-cultural adaptation component

#### Phase1: Translation of the SAS, PCPSS and palliative care phase

We translated the PCOC SAS, PCPSS, and Palliative Care Phase into Chinese according to the Brislin’s forward-and-backward translation model [15]. Initially, two proficient postgraduates (T1 and T2), majoring in Nursing and English Education, independently translated these tools into Chinese. Inconsistencies were resolved through discussion, resulting in a preliminary Chinese version. This version was then back-translated into English by T3, a proficient nurse unexposed to the tools. Two Australian PCOC academic staff with clinical backgrounds identified differences between the back-translated version and the original, any discrepancies were resolved through discuss among the three Chinese translators and the two PCOC staff. Finally, the Chinese-speaking research team finalized the Chinese version, addressing any ambiguities.

#### Phase 2: Cognitive interviews

Cognitive interviews [16] were undertaken to culturally adapt the three tools. As the PCPSS and Palliative Care Phase are clinician-rated tools, six Chinese palliative care clinicians (three nurses and three doctors) who had completed core education in the PCOC model (i.e., the PCOC fundamentals education sessions) were invited to assess palliative care inpatients using the two tools. As the PCOC-SAS is a patient-reported tool, five Chinese palliative care inpatients were invited to assess their symptom distress using the PCOC SAS (Supplementary Table 1: Participants characteristics for cognitive interviews). The “think-aloud” technique, which involves verbalizing thoughts while using the tools, and together with the “read-aloud” technique, which involves verbalizing thoughts while reading the items of the tools, were employed along with verbal probing (asking questions during tool use) to obtain feedback from the patients and clinicians regarding the tools’ clarity, comprehensibility, and interpretability [17] (Supplementary Table 2: Cognitive interview guideline). Field notes were taken during the interviews.

### Psychometric testing component

#### Study settings

The psychometric testing of the tools was conducted in the 20-bed palliative care unit and the 16-bed advanced cancer care unit at a Cancer Hospital in mainland China, where all patients with advanced cancer and palliative care in conjunction with active disease treatment were cared for.

#### Participants

Palliative care clinicians from both the palliative care (15 clinicians; three doctors, 12 nurses) and advanced cancer care units (*n* = 10 clinicians; eight nurses, two doctors), along with patients admitted to these units for symptom management were invited to participate in the study between February 2023 and April 2023.

#### Measurements

##### The PCOC symptom assessment scale (PCOC SAS)

The PCOC SAS is an 11-point scale that allows patients or proxies to rate patients’ level of distress associated with seven common physical symptoms (i.e. difficulties with sleep, appetite problems, nausea, bowels problems, breathing problems, fatigue and pain) during the previous 24 h [9]. The Cronbach’s alpha coefficient of PCOC SAS was 0.59 for patient ratings and 0.62 for patient and proxy ratings combined [9], and the Pearson’s correlation coefficient for test-retest reliability was moderate or substantial in Australian palliative care settings [9, 18].

##### Palliative care problem severity scale (PCPSS)

The PCPSS is a four-point, clinician-rated tool that assesses the severity (0-absent to 3-severe) of palliative care problems in four domains: pain, other symptoms, psychological/spiritual problems, and family/carer problems. In the Australian setting, it demonstrated moderate inter-rater reliability (weighted kappa = 0.38–0.48) [10].

##### Palliative care phase

The Palliative Care Phase is a clinician-rated tool that describes the patient’s clinical condition and their family/carers’ condition and informs the urgency and level of care required. It categorizes each patient’s condition into four non-sequential phases (‘stable’, ‘unstable’, ‘deteriorating’ and ‘terminal’) through comprehensive clinical assessments [8]. An acceptable level of inter-rater reliability (weighted Kappa = 0.67) and high acceptability were reported among Australian clinicians [11].

##### The Edmonton symptom assessment system (ESAS)

The Edmonton Symptom Assessment System (ESAS) allows palliative care patients to self-report the severity of 11 common symptoms using a 0–10 numerical scale [19]. The Chinese version of ESAS (C-ESAS) demonstrated acceptable internal consistency (Cronbach’s alpha = 0.72), strong test-retest reliability (*r* = 0.47–0.92), and good concurrent validity with the Chinese version of M.D. Anderson Symptom Inventory (*r* = 0.66–0.96) [20].

#### Sample size

We employed software PASS 15 to calculate the sample size. The weighted kappa statistic (*k*) was chosen to determine the significance of the level of agreement between clinicians [21]. We hypothesized that the weighted kappa coefficient would be similar to the Australian study where the overall agreement between palliative care professionals’ rating of Palliative Care Phase was 0.67 (95%CI = 0.61–0.70) [11]. We used the Palliative Care Phase for sample size determination given it yielded the largest sample size in contrast to other PCOC tools. To achieve 80% statistical power with a 95% confidence interval of 0.15, a minimum sample size of 298 paired assessments was required. Accounting for a 20% attrition rate due to patients not being assessed by two palliative care clinicians within 4 h, the sample size was inflated to 357 paired assessments.

#### Data collection

Two clinicians on the same shift in the same unit used the PCPSS and the Palliative Care Phase to assess the same inpatient. In parallel, patients rated their symptoms in relation to severity and distress using the C-ESAS and PCOC SAS respectively. Considering the potential variability in needs throughout the day for palliative care inpatients, the patient and two clinicians were required to complete the assessments within four hours to ensure consistency. The patients’ demographic information was collected from their medical records, and palliative care clinicians’ demographic details were gathered after their completion of the PCPSS and Palliative Care Phase assessments.

### Analysis

All analyses were performed using SPSS 25.0 [22]. Demographic information was reported using descriptive statistics. A significant *p* value was set at 5%. We assessed the reliability, validity, and sensitivity of the PCOC SAS, PCPSS and Palliative Care Phase using the following methods.

#### PCOC SAS

##### Validity

The **discriminant validity** of the PCOC SAS was assessed using the Spearman’s correlation coefficient between the PCOC SAS and the C-ESAS, and the PCOC SAS and PCPSS. Given the PCOC SAS is a patient-reported tool used to assess symptom distress, and PCPSS is a clinician-rated tool to examine symptom severity, and that clinicians tend to underestimate a range of patient’s symptoms [23, 24], we hypothesized that there would be a moderate correlation between PCOC SAS-pain and PCPSS-pain, as well as the PCPSS-other symptoms and the PCOC SAS total items (excluding the pain item) scores. We investigated the correlation between Chinese versions of PCOC SAS and C-ESAS. Both are patient-reported tools, but C-ESAS is to assess symptoms severity (or intensity) whereas the PCOC SAS assesses symptom-related distress. Therefore, we hypothesised that there would be a moderate correlation between the PCOC SAS pain and C-ESAS pain, PCOC SAS-tiredness and C-ESAS-fatigue (the same Chinese vocabulary was used to describe “tiredness” and “fatigue”), PCOC SAS nausea and C-ESAS nausea, PCOC SAS appetite and C-ESAS appetite problems; and the correlation between PCOC SAS breathing problems and C-ESAS-shortness of breath was degraded from moderate to low, as PCOC SAS breathing problems encompass shortness of breath and other breathing issues. For interpretation, we considered *r* = 0.30–0.49 as low correlation, *r* = 0.50–0.69 as moderate, *r* = 0.70–0.89 as high, *r* = 0.90-1.00 as very high [25].

##### Reliability

To evaluate the **internal consistency**, the Cronbach’s alpha was calculated, and a Cronbach’s alpha of ≥ 0.8 indicates signified good internal consistency [26].

#### PCPSS

##### Validity

The **concurrent validity** of the PCPSS was assessed by examining Spearman’s correlation coefficient between the PCPSS and the C-ESAS. Given PCPSS and C-ESAS are clinician-rated and patient-reported tools respectively, our hypotheses were that there would be a moderate correlation between PCPSS-pain and C-ESAS-pain scores, PCPSS-other symptoms score and the total summed score of all C-ESAS items (excluding pain, depression and anxiety); and low correlation between C-ESAS-depression and anxiety and PCPSS-psychological/spiritual problems as PCPSS-psychological/spiritual problems domain encompasses a broader spectrum of emotional disorders.

##### Reliability

Cronbach’s alpha was used to assess **internal consistency**. For the **inter-rater reliability**, we used the weighted kappa statistic (*k*) to determine the level of agreement between two raters. k = 0.00–0.20 indicates “Slight” agreement, k = 0.21–0.40 as “Fair”, k = 0.41–0.60 as “Moderate”, k = 0.61–0.80 as “Substantial”, k = 0.81–1. 00 as “Almost perfect” [27].

#### Palliative care phase

##### Reliability

The weighted kappa statistic (*k*) was used to assess the **inter-rater reliability**.

##### Sensitivity

The **sensitivity** of Palliative Care Phase was assessed by examining its capacity to predict the levels of severity of symptoms distress and symptoms in PCOC SAS and PCPSS, respectively. Informed by previous findings [9], our hypotheses were that patients in an unstable/deteriorating phase would exhibit higher levels of symptom distress on each PCOC SAS item and higher levels of symptom severity on each PCPSS domain, compared to those in stable phase, as changes to the care plan are needed when patients are in an unstable/deteriorating phase. The sensitivity of Palliative Care Phase was analyzed using Chi-square testing.

## Results

### Cross-cultural adaptation

Only minor grammatical and wording discrepancies were identified in the forward translations of the tools, and were easily resolved through discussion by the two translators. Cognitive interview results revealed a salient need to have a clear explanation about how symptom severity and Palliative Care Phase can be linked to the care plan. “My suggestion is that you need to clearly indicate the Palliative Care Phase and the rating score for the PCPSS are based on the care plan, as without this information, we are unsure how to assess the Phase and the symptoms severity for patients”, two nurses expressed their concerns. The think-aloud component of the cognitive interviewing also revealed that clinicians were concerned about how their clinical experience, palliative care capability, and their familiarity with patients might influence their assessments of the patient. “I am afraid that it will take us more time if we are not familiar with the patients, especially for those new patients who are just admitted to our ward”. Two nurses and one doctor concerned about the time spent on the PCOC assessment. “The clinicians may have different scores, as the ratings are linked to the care of plan, particularly the differences between a new staff and senior staff, this is because it is a challenge for a new staff to make the appropriate clinical judgment due to their palliative care knowledge and clinical experience”, a nurse concerned. Also, ambiguity regarding the concept of distress in relation to PCOC SAS was evident. “I don’t know the differences between symptom severity and symptom distress severity if you don’t explain it to me”. “I suggest you include the word “distress” at the top of the color and facial expression scale, or you highlight “distress” on the scale”. As a result, for the PCOC SAS, two changes were made to clarify that it assesses symptom distress rather than severity: (i) The name changed from “Symptom Assessment Scale” to “Symptom Distress Assessment Scale”; (ii) In the instruction section at the top of the color and facial expression scale, “Absent”, “Mild”, “Moderate” and “Severe” were revised to “Absent Distress”, “Mild Distress”, “Moderate Distress” and “Severe Distress” .

### Validation of the point-of-care outcomes assessment tools

#### Participants characteristics

Twenty two out of 25 clinicians from the palliative care and advanced cancer care units at a Cancer Hospital participated in this study. Most were nurses (90.9%) and female (95.5%). Years of experience varied, with 40.9% having worked ≥ five years in palliative care. A total of 368 paired assessments were completed for 135 inpatients. The average age of the inpatients was 59.4 (Table 1).

**Table 1:** Patient characteristics (*N* = 135)

Characteristics		N (%)
Age	< 65	85 (63.0)
	≥ 65	50 (37.0)
Gender	Male	85 (63.0)
	Female	50 (37.0)
Diagnosis	Lung cancer	53 (39.3)
	Colorectal cancer	23 (17.0)
	Oesophageal gastric cancer	22 (16.3)
	Hepatobiliary pancreatic cancer	14 (10.4)
	Oral cancer	5(3.7)
	Gynecological cancer	3 (2.2)
	Brain cancer	2 (1.5)
	Prostatic cancer	2 (1.5)
	Other cancers (including for example kidney, bone, pelvic, retroperitoneal, adrenal)	11 (8.1)

#### PCOC SAS

##### Internal consistency

The PCOC SAS demonstrated good internal consistency with a Cronbach’s Alpha of 0.85 (Supplementary Table 3).

##### Discriminant validity

As anticipated, a moderate correlation was observed between the scores of PCPSS-other symptoms and the total summed score of all PCOC SAS items excluding the pain item (*r* = 0.56, *P* < 0.001). A strong correlation was found between PCOC SAS-pain and PCPSS-pain (*r* = 0.79, *P* < 0.001). Regarding the correlation between related symptoms on PCOC SAS and C-ESAS, the results indicated low correlations for the three corresponding symptoms: Nausea (*r* = 0.47, *P* < 0.001), breathing problems (*r* = 0.38, *P* < 0.001) and fatigue (*r* = 0.41, *P* < 0.001), and moderate correlations were found for the items “pain” (*r* = 0.51, *P* < 0.001) and “appetite” (*r* = 0.51, *P* < 0.001).

#### PCPSS

##### Internal consistency

The PCPSS demonstrated acceptable internal consistency with a Cronbach’s Alpha of 0.75 (Supplementary Table 4).

##### Inter-rater reliability

The number of paired assessments varied between 360 and 365 for each domain of the PCPSS. The largest proportion matched were evidenced for pain (64.7%), followed by family/carer problems (52.9%), other symptoms (51.7%) and psychological/spiritual problems (44.8%) (Table 2). Among the four domains of PCPSS, only the domain of pain achieved a moderate strength of agreement, whereas the other three domains exhibited a fair strength of agreement (Table 3). The largest proportion of discordant ratings were observed between assessments of mild and moderate within the domain of pain, and assessments of absent and mild within the remaining three domains (Table 2).

**Table 2:** The inter-rater rating characteristics for PCPSS N (%)

	Rating		Pain (*N* = 365)	Other symptoms (*N* = 360)	Psychological/spiritual (*N* = 362)	Family/carer (*N* = 365)
	Rater 1	Rater 2				
Matched ratings	Absent	Absent	102 (27.9)	51 (14.2)	30 (8.3)	82 (22.5)
	Mild	Mild	101 (27.7)	103 (28.6)	93 (25.7)	87 (23.8)
	Moderate	Moderate	32 (8.8)	31 (8.6)	39 (10.8)	23 (6.3)
	Severe	Severe	1 (0.3)	1 (0.3)	0 (0)	1 (0.3)
	**Total matched**		**236 (64.7)**	**186 (51.7)**	**162 (44.8)**	**193 (52.9)**
Mismatched ratings	Absent	Mild	26 (7.1)	54 (15.0)	60 (16.6)	75 (20.5)
	Absent	Moderate	10 (2.7)	15 (4.2)	6 (1.7)	13 (3.6)
	Absent	Severe	1 (0.3)	2 (0.6)	0 (0)	0 (0)
	Mild	Absent	15 (4.1)	42 (11.7)	52 (14.4)	44 (12.1)
	Mild	Moderate	27 (7.4)	24 (6.7)	39 (10.8)	18 (4.9)
	Mild	Severe	2 (0.5)	1 (0.3)	2 (0.6)	0 (0)
	Moderate	Absent	2 (0.5)	4 (1.1)	3 (0.8)	4 (1.1)
	Moderate	Mild	36 (9.9)	25 (6.9)	29 (8.0)	17 (4.7)
	Moderate	Severe	2 (0.5)	2 (0.6)	0 (0)	0 (0)
	Severe	Absent	0 (0)	0 (0)	0 (0)	1 (0.3)
	Severe	Mild	1 (0.3)	1 (0.3)	4 (1.1)	0 (0)
	Severe	Moderate	7 (1.9)	4 (1.1)	5 (1.4)	0 (0)
	**Total mismatched**		**129 (35.3)**	**174 (48.3)**	**200 (55.2)**	**172 (47.1)**

**Table 3:** The Inter - rater agreement for PCPSS

PCPSS domains	Weighted Kappa (k)	95% confidence interval	P	Strength of agreement	Agreement
Pain	0.53	0.46–0.59	< 0.001	Moderate	64.6%
Other symptoms	0.30	0.22–0.38	< 0.001	Fair	51.7%
Psychological/spiritual problems	0.23	0.15–0.30	< 0.001	Fair	44.8%
Family/carer problems	0.29	0.21–0.38	< 0.001	Fair	52.9%

##### Concurrent validity

A moderate correlation was found in “pain” item between the PCPSS and C-ESAS (*r* = 0.52, *P* < 0.001), and a low correlation was observed between PCPSS-psychological/spiritual problems and C-ESAS-depression and anxiety (*r* = 0.38, *P* < 0.001). A low correlation was observed between PCPSS-other symptoms and all C-ESAS symptoms excluding pain, depression, and anxiety (*r* = 0.37, *P* < 0.001).

#### Palliative Care Phase

##### Inter-rater reliability

A fair strength of agreement (Weighted Kappa = 0.31, 95% CI = 0.17–0.45, *p* < 0.001) was achieved between two raters when using the Palliative Care Phase. The most mismatched assessments were for stable/deteriorating phase, accounting for most mismatched (*n* = 32/59 mismatched or 62.7%) (Table 4).

**Table 4:** The inter-rater rating characteristics for Palliative Care Phase (*N* = 334 paired assessments)

	Raters		N (%)
	1	2	
Matched ratings	Stable	Stable	262 (78.4)
	Unstable	Unstable	4 (1.2)
	Deteriorating	Deteriorating	8 (2.4)
	Terminal	Terminal	1 (0.3)
	**Total matched**		**275 (82.3)**
Mismatched ratings	Stable	Unstable	5 (1.5)
	Stable	Deteriorating	18 (5.4)
	Unstable	Stable	12 (3.6)
	Unstable	Deteriorating	1(0.3)
	Deteriorating	Stable	19(5.7)
	Deteriorating	Unstable	2(0.6)
	Deteriorating	Terminal	2(0.6)
	**Total mismatched**		**59 (17.7)**

##### Sensitivity

The results were consistent with our hypothesis that patients in unstable/deteriorating phase reported higher levels of symptom distress (Table 5). Similarly, patients in unstable/deteriorating phase had higher levels of symptom severity (Table 6).

**Table 5:** The sensitivity of the Palliative Care Phase in predicting the levels of symptom distress severity

PCOC SAS Symptom distress level		Palliative Care Phase N (%)			*P*
		Stable	Unstable	Deteriorating	
Sleeping (*N* = 351)	Absent/Mild	268 (76.4)	7 (2.0)	19 (5.4)	< 0.001
	Moderate/Severe	32 (9.1)	10 (2.8)	15 (4.3)	
Appetite (*N* = 351)	Absent/Mild	263 (74.9)	13 (3.7)	21 (6.0)	< 0.001
	Moderate/Severe	37 (10.5)	4 (1.1)	13 (3.7)	
Nausea (*N* = 350)	Absent/Mild	284 (81.1)	15 (4.3)	27 (7.7)	0.02
	Moderate/Severe	16 (4.6)	2 (0.6)	6 (1.7)	
Bowels (*N* = 351)	Absent/Mild	275 (78.3)	14 (4.0)	21 (6.0)	< 0.001
	Moderate/Severe	25 (7.1)	3 (0.9)	13 (3.7)	
Breathing (*N* = 351)	Absent/Mild	290 (82.6)	13 (3.7)	29 (8.3)	< 0.001
	Moderate/Severe	10 (2.8)	4 (1.1)	5 (1.4)	
Fatigue (*N* = 351)	Absent/Mild	278 (79.2)	12 (3.4)	22 (6.3)	< 0.001
	Moderate/Severe	22 (6.3)	5 (1.4)	12 (3.4)	
Pain (*N* = 351)	Absent/Mild	275 (78.3)	11 (3.1)	17 (4.8)	< 0.001
	Moderate/Severe	25 (7.1)	6 (1.7)	17 (4.8)	

*Note* Terminal phase excluded due to only one patient assessed as being within the terminal phase

**Table 6:** The sensitivity of the Palliative Care Phase in predicting the levels of symptom severity

PCPSS Symptom severity level		Palliative Care Phase N (%)			*P*
		Stable	Unstable	Deteriorating	
Pain (*N* = 354)	Absent/Mild	253 (71.5)	6 (1.7)	18 (5.1)	< 0.001
	Moderate/Severe	49 (13.8)	11 (3.1)	17 (4.8)	
Other symptoms (*N* = 353)	Absent/Mild	261 (73.9)	6 (1.7)	21 (5.9)	< 0.001
	Moderate/Severe	40 (11.3)	11 (3.1)	14 (4.0)	
Psychological/spiritual problems (*N* = 353)	Absent/Mild	258 (73.1)	7 (2.0)	13 (3.7)	< 0.001
	Moderate/Severe	43 (12.2)	10 (2.8)	22 (6.2)	
Family/carer problems (*N* = 354)	Absent/Mild	284 (80.2)	9 (2.5)	21 (5.9)	< 0.001
	Moderate/Severe	18 (5.1)	8 (2.3)	14 (4.0)	

*Note* Terminal phase excluded due to only one patient assessed as being within the terminal phase

## Discussion

To our knowledge, this study is the first to examine cross-cultural adaptation of the PCOC SAS, PCPSS and Palliative Care Phase from the PCOC quality program and to assess their validity in the Chinese context. As informed by the cognitive testing results, cross-cultural adaptation highlighted the necessity of clarifying the link between the patient’s care plan and the assessment information, and the need to further clarify the concept of distress for the PCOC SAS. In relation to the psychometric testing our findings indicated good internal consistency for the Chinese version of PCOC SAS and acceptable consistency for PCPSS. The moderate/low correlation between corresponding items in the PCOC SAS and PCPSS, PCOC SAS and C-ESAS, as well as PCPSS and C-ESAS were consistent with our expectations. Discriminant validity for PCOC SAS and concurrent validity for PCPSS were good/fair. Palliative Care Phase was associated with the levels of symptom distress and symptom severity for palliative care patients. Acceptable levels of inter-rater reliability were demonstrated for both PCPSS and Palliative Care Phase.

The PCPSS exhibited acceptable consistency with a Cronbach’s Alpha of 0.75, slightly below the conventional threshold for good internal consistency (0.8). This may be attributed to the four domains of the PCPSS not being highly correlated with each other. Despite not being highly correlated, these four domains (pain, other physical symptoms, psychological/spiritual problems, family/carer problems) are considered the optimal and pivotal aspects in the context of palliative care, serving as crucial components for a comprehensive assessment [28]. Additionally, having only one item within each domain may contribute to the relatively lower Cronbach’s Alpha [29].

In our study, we found that the item pain on both PCOC SAS and PCPSS demonstrated strong correlation, contrary to our initial hypothesis of only a moderate correlation. However, our result was consistent with the validation study for the PCOC SAS in the Australian palliative care settings [9]. Our study also revealed a moderate correlation regarding pain assessed with PCPSS and C-ESAS, and moderate correlation regarding pain between PCOC SAS and C-ESAS. Pain is one of the most distressing symptoms experienced by patients, and its severity often leads to a higher level of distress [30]. Given the significance of pain management in palliative care, our findings highlight the importance of future research focusing on verifying the relationship between assessments of the severity of pain and associated distress. Understanding this relationship could lead to more effective pain management for palliative care patients. For example, pain may only need to be assessed on PCOC SAS or PCPSS if the pain severity and pain distress were closely related.

The most mismatched ratings for the clinician-rated Palliative Care Phase were stable/deteriorating, constituting 6.8% of mismatched scores out of the 290 instances of deteriorating or stable phases. The largest proportion of discordant ratings for PCPSS were mild/moderate within the domain of pain, and absent/mild within the remaining three domains. These mismatched ratings occurred less commonly for unstable phase, or where symptoms were severe, which indicated that clinicians may find it easier to recognize and address urgent needs in patients. It may also indicate the risk of earlier, escalating needs being missed.

In Australian palliative care settings, moderate agreement levels were observed for both Palliative Care Phase (k = 0.67, 95% CI = 0.61–0.70) [11] and PCPSS (k = 0.40–0.48, 95%CI = 0.036–0.54), except for the domain of other symptoms (k = 0.38, 95%CI = 0.32–0.45) [10]. Since the PCOC model has been used in Australian since 2005, most Australian palliative care professionals had received comprehensive training and had extensive experience in its application by the time of that study. However, it is the first time the PCOC model has been introduced in a Mainland Chinese hospital, where only a fundamental training session on how to use the PCOC assessment tools was offered to Chinese palliative care providers, and our cognitive interviewing results showed that when using the tools the clinicians questioned their clinical experience, palliative care capability, and their familiarity with patients and how this might influence their assessments. Given that palliative care started gaining attention in Mainland China only in 2017 (six years prior to this study), it is understandable that Chinese palliative care providers have such concerns, as the PCOC assessment tools are not used in isolation for data collection but serve as tools to inform the patient’s care plan. Similarly, Australian palliative care providers have mentioned feeling more confident in assessing patients’ problem severity and palliative care phase when they were familiar with the patient’s clinical condition [10, 11]. Consequently, further education sessions regarding the PCOC model are needed for Chinese palliative care providers or alternatively modifications to improve the validity of the measures are required.

## Limitations

Study limitations include that the participants were from two wards within a single hospital and were cancer patients only. Future studies should encompass diverse settings and diagnoses. Attempting test-retest reliability for PCOC SAS for twice a day led to patient annoyance, suggesting longer re-test intervals might be more acceptable. Additionally, the weighted Kappa statistic is affected by the prevalence of the results [31]. Hence, the Stable phase was more prevalent in this study, yielding a fair agreement (weighted kappa coefficient of 0.31) despite a high percentage of matched ratings (82.3%) for Palliative Care Phase. For PCPSS, while matched ratings were lower for other symptoms (51.7%) than for family/carer (52.9%), the weighted kappa coefficient was higher (k = 0.30, 95% CI = 0.22–0.38 vs. k = 0.29, 95% CI = 0.21–0.38), and given this we recommend caution when interpreting this finding.

## Conclusions

Our preliminary study has shown that the PCOC measures are useful for routine clinical care in palliative care in China. The Chinese version of PCOC SAS demonstrate good internal consistency, and the PCPSS showed fair levels. Palliative Care Phase proved sensitive in detecting in patients’ clinical acuity and urgency of needs. However, to enhance scoring reliability among clinicians, education and training in the measures are necessary.

### Electronic supplementary material

Below is the link to the electronic supplementary material.

Supplementary Material 1

## Data Availability

The datasets used and/or analysed during this study are available from the corresponding author on reasonable request.

## Abbreviations

PCOC: Palliative Care Outcome Collaboration
PCOC SAS: The PCOC Symptom Assessment Scale
PCPSS: Palliative Care Problem Severity Scale
C-ESAS: Chinese version of Edmonton Symptom Assessment System
AKPS: Australia-modified Karnofsky Performance Status
RUG-ADL: Resource Utilization Groups - Activities of Daily Living
*K*: Weighted kappa statistic
