# Supplementary material for: Cross-cultural adaptation and psychometric validation of point-of-care outcome assessment tools in Chinese palliative care clinical practice
Source: BMC Palliat Care. 2024 Apr 3;23:89. doi: 10.1186/s12904-024-01395-6 (PMC10988912; doi:10.1186/s12904-024-01395-6)
Supplement: Supplementary file 1 — Supplementary Material 1 [file 12904_2024_1395_MOESM1_ESM.docx]

**Supplementary table 1 Participants characteristics for cognitive interview**

| **Participants characteristics** | | **N** |
| --- | --- | --- |
| **Clinicians (N=6, cognitive interview for PCPSS and Palliative Care Phase)** | | |
| Age (Mean, Range) | | 32.0 (29-35) |
| Gender | Male | 1 |
|  | Female | 5 |
| Occupation | Doctor | 3 |
|  | Nurse | 3 |
| Work experience in palliative care (Year) | <5 | 2 |
|  | ≥5 | 4 |
| PCOC training | Yes | 6 |
|  | No | 0 |
| **Palliative care patients (N=5, cognitive interview for PCOC SAS)** | | |
| Age (Mean, Range) | | 46.5 (26-61) |
| Gender | Male | 3 |
|  | Female | 2 |
| Educational level | Elementary school | 2 |
|  | Middle and high school | 2 |
|  | Bachelor’s degree and above | 1 |
| Diagnosis | Oral cancer | 1 |
|  | Bone cancer | 1 |
|  | Lung cancer | 1 |
|  | Colorectal cancer | 1 |
|  | Brain cancer | 1 |

**Supplementary table 2 Questions for the cognitive interviews**

| **PCPSS and Palliative Care Phase (For palliative care clinicians)** |
| --- |
| 1. Do you find the definition/instruction for PCPSS/Palliative Care Phase easy to understand? |
| 1. Is it difficult for you to assess patients by using PCPSS/Palliative Care Phase? |
| 1. Are there some parts of PCPSS/Palliative Care Phase that need to be modified? |
| 1. Do you think PCPSS/Palliative Care Phase is useful in guiding your clinical practice? |
| **PCOC SAS (For palliative care patients)** |
| 1. Is it difficult for you to assess your symptom distress by using the PCOC SAS? |
| 2. Are there some parts of the PCOC SAS that need to be modified? |

**Supplementary table 3 Internal consistency of PCOC SAS (N= 364 paired assessments)**

| **PCOC SAS Items** | **Symptoms distress level N (%)** | | | | **Cronbach’s Alpha** |
| --- | --- | --- | --- | --- | --- |
|  | **Absent** | **Mild** | **Moderate** | **Severe** |  |
| Sleeping problems | 93 (25.5) | 211 (58.0) | 49 (13.5) | 11 (3.0) | 0.85 |
| Appetite problems | 115 (31.6) | 193 (53.0) | 42 (11.5) | 14 (3.8) |  |
| Nausea | 194 (53.3) | 144 (39.6) | 19 (5.2) | 7 (1.9) |  |
| Bowels problems | 168 (46.2) | 154 (42.3) | 32 (8.8) | 10 (2.7) |  |
| Breathing problems | 215 (59.1) | 125 (34.3) | 20 (5.5) | 4 (1.1) |  |
| Fatigue | 83 (22.8) | 241 (66.2) | 36 (9.9) | 4 (1.1) |  |
| Pain | 123 (33.8) | 187 (51.4) | 47 (12.9) | 7 (1.9) |  |
| Other symptoms | 182 (50.0) | 164 (45.1) | 15 (4.1) | 3 (0.8) |  |

**Supplementary table 4 Internal consistency of PCPSS (N=365 paired assessments)**

| **PCPSS Item** | **Symptoms severe level N (%)** | | | | **Cronbach’s Alpha** |
| --- | --- | --- | --- | --- | --- |
|  | **Absent** | **Mild** | **Moderate** | **Severe** |  |
| Pain | 140 (37.8) | 147 (39.7) | 73 (19.7) | 9 (2.4) | 0.75 |
| Other symptoms | 123 (33.2) | 175 (47.3) | 64 (17.3) | 16 (1.6) |  |
| Psychological/spiritual | 97 (26.2) | 188 (50.8) | 73 (19.7) | 9 (2.4) |  |
| Family/carer | 174 (47.0) | 149 (40.3) | 44 (11.9) | 2 (0.5) |  |

**
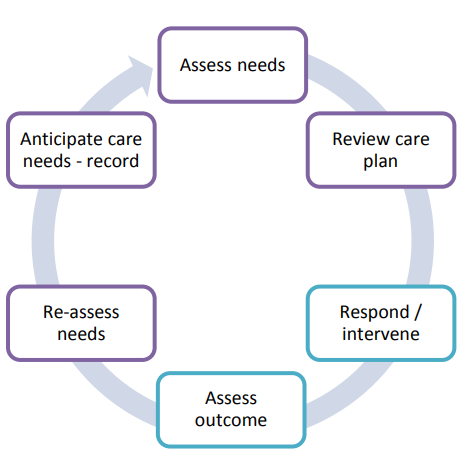
**

**Supplementary figure 1 PCOC routine clinical assessment and response framework**

**
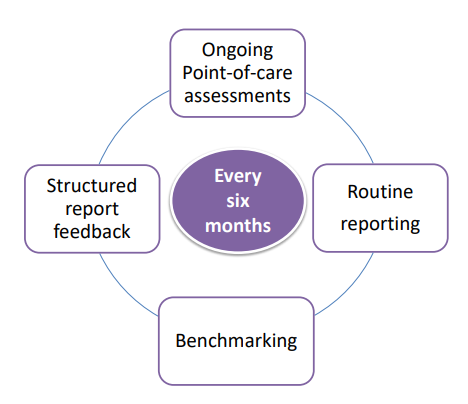
**

**Supplementary figure 2 PCOC cycle**

**The supplementary file I**

**PCOC评估量表 (PCOC assessment tools)**

| **患者评分Patient rated score** | **症状困扰度评估量表（Symptom Distress Assessment Scale, SDAS)**  **评分范围：0-10.**0=没有困扰，10=最严重程度的困扰  Range: 0-10. *0 = absent 10 = worst possible* | |
| --- | --- | --- |
|  | 睡眠问题Difficulty sleeping |  |
|  | 食欲问题Appetite |  |
|  | 恶心Nausea |  |
|  | 大小便问题Bowels |  |
|  | 呼吸问题Breathing |  |
|  | 疲乏Fatigue |  |
|  | 疼痛Pain |  |
|  | 其它症状Other |  |
| **医护人员评分**  **Clinician rated score** | **安宁疗护症状严重程度评估量表（Palliative Care Problems Severity Scale, PCPSS）**  **评分范围：0-3. 0=没有； 1=轻度； 2=中度； 3=重度**  **Range: 0-3. 0=Absent； 1=Mild； 2=Moderate； 3=Severe** | |
|  | 疼痛Pain |  |
|  | 其他症状Other Symptoms |  |
|  | 心理/精神问题Psychological / Spiritual |  |
|  | 家属/照顾者相关问题Family / Carer |  |
|  | **安宁疗护阶段量表（Palliative Care Phase）**  **1.稳定阶段**=监测 **2. 不稳定阶段**=需紧急处理 **3.恶化阶段**=对照护计划进行审查，评估照护计划是否有效 **4. 终末阶段**=提供临终照护服务  **1.Stable =** Monitor 2. **Unstable =** Urgent action required 3. **Deteriorating =** Review plan of care  4. **Terminal =** Provide EOL care | |
|  | 安宁疗护阶段 Palliative Care Phase |  |

**PCOC评估量表使用说明 (PCOC assessment tools manual)**

| **症状困扰度评估量表 （SDAS）** |
| --- |
| 症状困扰评估量表用来评估躯体症状对患者造成的困扰程度。主要包括7个最常见的症状。Patient Rated distress relating to symptoms over a 24hr period. The symptoms and problems in the scale are the seven most common.  **使用方法Usage：**   1. 最佳的评估方法为患者自行或在医护人员/家属/照顾者的协助下使用症状困扰评估量表进行自我评估。在患者没有交流能力或表达其症状困扰程度困难时（如昏迷、谵妄、终末阶段），该评估也可由代理人（如家属、照护者或医护人员）完成。Best practice is for the patient to rate distress either independent or with the assistance of a clinician or family/carer using a visual of the scale such as the *Symptom Assessment Scale Form for Patients*. Symptom distress may be rated by proxy. This only occurs when the patient is unable to participate in conversation relating to symptom distress i.e. Terminal phase. 2. 患者回顾其过去24小时内经历的躯体症状或问题，根据症状对其造成的困扰严重程度进行分级：**0分=没有；1-3分=轻度；4-7分=中度；8-10分=重度。**patient to consider their experience of the individual symptom or problem over the last 24 hours and rate distress into four levels: **0=Absent; 1-3=Mild; 4-7=Moderate; 8-10=Severe.**   **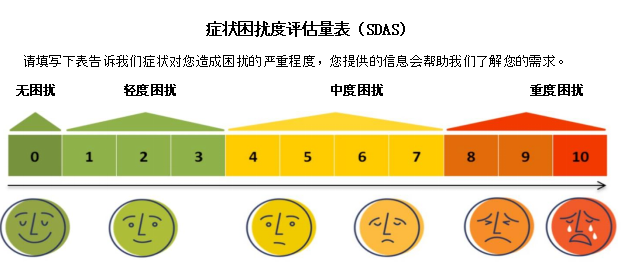**0=继续原照护计划；1-3=继续监测和记录患者情况；4-7=更改照护计划、会诊或其它医疗团队加入照护计划或实施干预措施；8-10=紧急处理。0 = Continue care; 1 -3 = Monitor and record ; 4-7 = Review/change plan of care; referral, intervention as required ; 8-10 = Urgent action. |
| **安宁疗护症状严重程度评估量表 （PCPSS）** |
| 1.医护人员通过评估患者过去24小时内症状/问题的严重程度，以帮助明确患者安宁照护的需求和制定合理的照护计划。Clinician rated assessment of problems over a 24hr period. Global assessment of four palliative care domains to summarise palliative care needs and plan care.  **疼痛：**患者疼痛问题的总体严重程度 **Pain:** overall severity of pain problems for the patient  **其他症状**：除了疼痛外其他一项或多项症状的总体严重程度**Other Symptoms:** overall severity of problems relating to one or more symptoms other than pain  **心理/精神问题：**患者一项或者多项心理/精神方面问题的严重程度。**Psychological / Spiritual:** severity of problems relating to the patient’s psychological or spiritual wellbeing. May be one or more issues.  **家属/照顾者相关问题**：家属/照顾者相关问题需与患者的情况或安宁疗护的需求有关系。可以根据书面、口头或观察的信息进行评估，无需家属/照顾者在场进行评估。**Family / Carer:** problems associated with a patient’s condition or palliative care needs. Family / Carer do not need to be present to asses needs as written, verbal or observational information may be used.  2.患者安宁疗护症状严重程度的评分范围为0-3分，**0=没有；1=轻度；2=中度；3=重度**The severity of problems are rated and responded to following using the scale:  **0 = Absent; 1 = Mild; 2 = Moderate; 3 = Severe**  它与照护计划直接相关：0=继续原照护计划；1=继续监测和记录患者情况；2=更改照护计划、会诊或其它医疗团队加入照护计划或实施干预措施；3=紧急处理。0 = Continue care; 1= Monitor and record ; 2 = Review/change plan of care; referral, intervention as required ; 3 = Urgent action. |
| **安宁疗护阶段(Palliative Care Phase)** |
| **稳定阶段：**目前所采取的照护措施能有效管控患者的症状和问题。继续监测患者需求、审查照护计划、预期患者可能的变化并及时应对。**Stable:** Symptoms and problems are adequately controlled by established management. Monitor, review, anticipate & respond.  **不稳定阶段**：患者出现新的问题，或患者现存的问题、或家属/照顾者问题的迅速恶化，需要马上更改照护计划或进行紧急治疗。需采取紧急应对措施。**Unstable:** An urgent change in the plan of care or emergency treatment is required due to development of a new problem &/or a rapid increase in the severity of existing problems &/or family/carer problems. Urgent response required.  **恶化阶段：**患者的功能状态不断的下降，和/或现有症状不断的恶化，和/或出现了预期范围内的新问题，和/或家属/照顾者出现了预期范围内的新问题。照护计划正在尽力解决这些预期的问题但仍需对患者进行定期评估。审查和更改照护计划。**Deteriorating:** The plan of care is addressing anticipated needs but requires periodic review due to gradual functional decline &/or worsening of existing symptoms &/or the development of new but expected problems &/or family/carer problems. Review & change care plan.  **终末阶段：**患者可能在近日内死亡。提供临终照护。**Terminal:** Death likely in a matter of days. Monitor, review & respond. |
